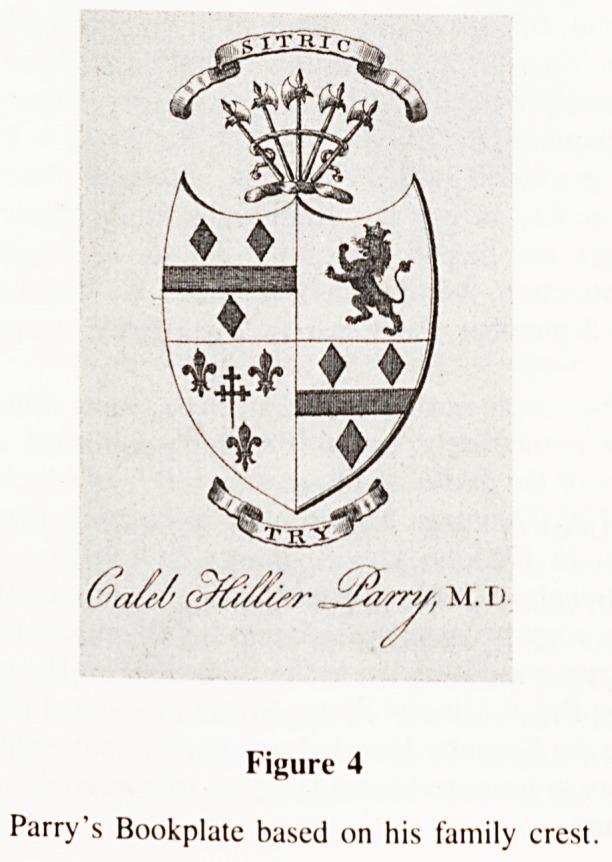# Caleb Hillier Parry

**Published:** 1991-12

**Authors:** Brian Jones


West of England Medical Journal Volume 106 (iv) December 1991

### Caleb Hillier Parry

**Brian Jones, BA, FLA**

**Fig. 1.**
Caleb Hillier Parry, MD, FRS. Steel-engraved portrait by Philip Audibert, after a sketch by John Hay Bell, 1804. Repr. from Joll, C.A., *Diseases of the thyroid gland,* London, Heinemann, 1932, front.

Dr. Caleb Hillier Parry, 1755-1822, is remembered as a distinguished Bath physician, original describer of several syndromes, geologist and eminent agriculturalist, creator of the Parry Library, and close friend and collaborator of Edward Jenner. Scion of a landed family in Pembrokeshire, Parry began his education at a private school in Cirencester, where he first met his lifelong friend, Edward Jenner. After three years at the Dissenters' Academy in Warrington, he became in 1773 a medical student at Edinburgh under William Cullen, moving two years later to London as a house-pupil of Dr. Thomas Denman. Returning to Edinburgh, Parry then graduated in 1778 with an inaugural dissertation on rabies. Before this, he was elected President of the Medical Society of Edinburgh, and helped to gain the Society its Royal Charter, a unique distinction. Admitted a licentiate of the Royal Society of Medicine in September, Parry married Miss Rigby, a noted beauty, in October.

In 1779 the couple settled in 13 Catherine Place, Bath. Success did not come immediately, and in 1781 Parry published *'Proposals for a history of the fossils of Gloucestershire'*, of which he left a collection of 20,000 items. A substantial MS existed, but remained unpublished. In 1785 he advised Jenner how to fly a hydrogen balloon at Berkeley Castle, only two years after Montgolfier's first flight. Parry soon became Physician to the Puerperal Charity, in 1782 Governor and Physician to the Bath General Hospital, later known as the Royal Mineral Water Hospital, and in 1789 the first Physician to the Casualty Hospital, which later merged with Bath City Infirmary to form the United Hospital, of which his son became first Physician.

From 1788 to 1793 Parry was an enthusiastic member of the Gloucestershire Medical Society, consisting of Parry, Jenner and three old friends, which met three times a year at the Fleece Inn at Rodborough, and the Minutes of which show the symbiotic relationship of Parry and Jenner at this time and the interplay of their ideas on vaccination and, particularly, on cardiology, a subject on which both addressed the Society. Jenner early described the 'effects produced on the vessels about the head by mechanical obstructions to the passage of the blood through the cavities of the heart', which can be linked with the paper read by Parry to the Medical Society of London in January 1789 'On the effects of compression of the arteries in various disorders, and in particular in those of the head'. Eventually published in 1792, the paper contains the first notice of the relief of pain given by carotid compression in hemicrania. In July 1788 Parry first read to the Society the work later published in 1799 as *'An Inquiry into the symptoms and causes of the Syncope Anginosa'*, and in July 1789 Jenner read some 'Remarks on a disease of the heart following acute rheumatism'. The success of the members' practices eventually killed the Society; in 1780 Parry made only £40 but by 1788 he made £1534 and later £300-£600 a month.

Celebrated as a doctor, Parry was at least as well known as an agriculturalist and a breeder of merino sheep, which he crossed with native breeds to improve their wool. In 1800 he published *'Facts and observations tending to show the practicability and advantage of producing in the British Isles clothing wool equal to that of Spain'*, to which he owed his election in the same year to the Fellowship of the Royal Society. In 1807 his *'An essay on the nature, produce, origin and extension of the merino breed of sheep'*, gained him a premium of £50 from the Board of Agriculture and honorary membership of the Farming Society of Ireland. By 1814 he was Vice-president of the Merino Society of London and a member of the Society of Natural History of Gottingen. His work was also noticed by George III, "Farmer George", who sent him two pure-bred merino rams from his own flock.

AN

INQUIRY

INTO THE

SYMPTOMS AND CAUSES

OF THE

SYNCOPE ANGINOSA,

COMMONLY CALLED

ANGINA PECTORIS;

ILLUSTRATED BY DISSECTIONS:

BY

CALEB HILLIER PARRY, M.D.

MEMBER OF THE COLLEGE OF PHYSICIANS OF LONDON, AND OF THE ROYAL MEDICAL SOCIETY OF EDINBURGH;

AND ONE OF THE PHYSICIANS OF THE BATH GENERAL HOSPITAL.

BATH, PRINTED BY R. CRUTTWELL;

AND SOLD BY

CADELL AND DAVIES, STRAND, LONDON.

1799.

**Fig. 2.**

Parry, C.H., *An Inquiry into the symptoms and causes of the Syncope Anginosa, commonly called Angina Pectoris, illustrated by dissections,* London, Davies; or Bath, Cruttwell, 1799, front.

In 1798 Edward Jenner published his *'Inquiry into the causes and effects of the variolae vaccinae . . .'*, dedicated to "My dear friend" C. H. Parry, M.D.; of the two preliminary drafts of the *'Inquiry'*, one, dated March 1797, has some pencilled suggestions in Parry's hand. Jenner's *'Further observations . . .'*, published in 1799, are also dedicated to the same "dear friend". In 1799 also, Parry published his own *'Inquiry', 'into the symptoms and causes of the Syncope Anginosa, commonly called Angina Pectoris'*. The classic description of angina William Heberden's, but Parry's book is considered the first publication in which the syndrome was associated with coronary heart disease. The book was inspired, as Parry freely admits, by previous observations of Jenner's. In 1778 Jenner had written to Heberden suggesting that John Hunter's anginal attacks were due to coronary artery ossification that Jenner had noted. This letter was never sent, but Jenner wrote similarly to Parry in 1785, and Parry brought his view to the Fleece Society, and then to the public.

**Fig. 3.**

Parry's residence at No. 27, The Circus.

Now a respected and prosperous doctor and scientist, Parry moved his residence in 1800 to 27 The Circus, where a memorial plaque was erected in 1925 when the B.M.A. visited Bath. His son notes that "nearly the whole catalogue of British nobility and many of the most distinguished men of the kingdom visited Bath for his advice". He was certainly now a very busy man.

In 1814 Parry returned to the subject of his inaugural dissertation in his *'Cases of tetanus and rabies contagiosa, or canine hydrophobia'*, dedicated to Parry's "dear and oldest friend", Jenner, in celebration of "our mutual friendship, uninterrupted during nearly 50 years". Parry describes three cases, including one of hysterical mimicry, and suggests public health measures. Over the years, Parry took detailed case notes intended for a comprehensive work, *'Elements of pathology and therapeutics'*, but he himself completed only the first volume, on *'General pathology'*, which appeared in 1815. Parry remained interested in the arteries and blood supply, and in 1816 published *'An experimental Inquiry into the nature, cause and varieties of the arterial pulse . . .'* About 30 vivisections on sheep or rabbits led him to conclude that the pulse wave is due to the impulse given by the systole of the left ventricle, and not to active contraction and relaxation of the arterial wall. In the second part of the book, he describes how, after a sheep's carotid has been obliterated, new vessels come to unite the two blind ends, contending though that these are new formations and not expansions of existing channels.

In October 1816 Parry suffered a paralytic stroke and retired to 7 Sion Hill Place, dying on the 9th March 1822 aged 66; his funeral, according to the *'Bath Chronicle'*, "gave rise to scenes never before seen in Bath". Edward Jenner was one of the pall-bearers; he died himself in the following year. Parry was buried in Bath Abbey, where a marble slab marks the spot. A separate memorial tablet was erected by his professional colleagues; the books with which it is adorned are also a worthy memorial to Parry's Library, as is the family crest, which he used as a bookplate.

Parry's considerable library passed to his son Charles Henry, who 23 years later presented it in 1845 to the United Hospital. The collection then languished in a damp basement until in 1933 it was deposited for safe keeping in the City Reference Library, where it was first catalogued and classified. In 1948 the collection was gifted by the Board of Management of the R.U.H to the City of Bath, together with some 220 items added by Dr. John Soden, and two years later, in 1950, the entire medical collection was presented to Bristol University Medical Library. Many of the volumes were by this time in very poor condition, and the University, with help from the Wellcome Trust, spent almost £4000 on repairs. The Parry Library proper now amounts to some 900 volumes, shelved in glazed cupboards in the Medical History Room of the Medical Library.

Perhaps the most interesting of Parry's works is the posthumous *'Collections from the unpublished medical writings'* published by his son, Charles Henry, in 1825. Parry had an excellent eye for neglected syndromes: the book contains one of the earliest descriptions of mitral stenosis, of facial hemiatrophy and "histaminic cephalgia"; and Parry gives a vivid description of megacolon twenty years before Ammon. Probably most important, Parry gives a remarkable original description of exophthalmic goitre. An outstanding example of multiple eponyms, the disease has been named at various times after nine describers, but one has to agree with Sir William Osler that if any man's name should be associated with exophthalmic goitre, "undoubtedly it should be that of the distinguished old Bath physician".

**Figure 4**

Parry's Bookplate based on his family crest.

## Figures and Tables

**Fig. 1. f1:**
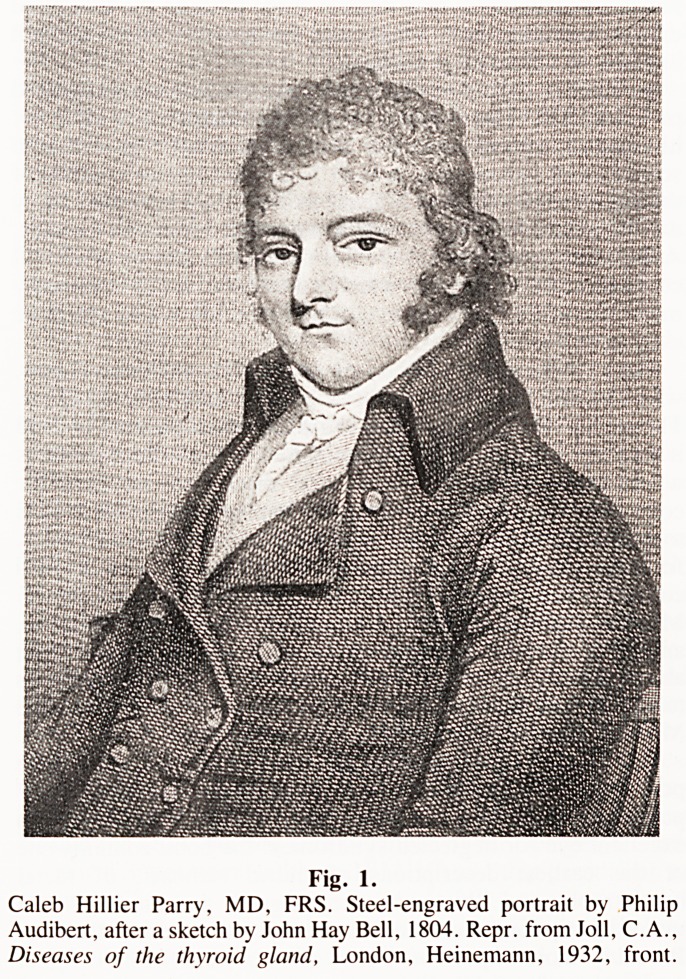


**Fig. 2. f2:**
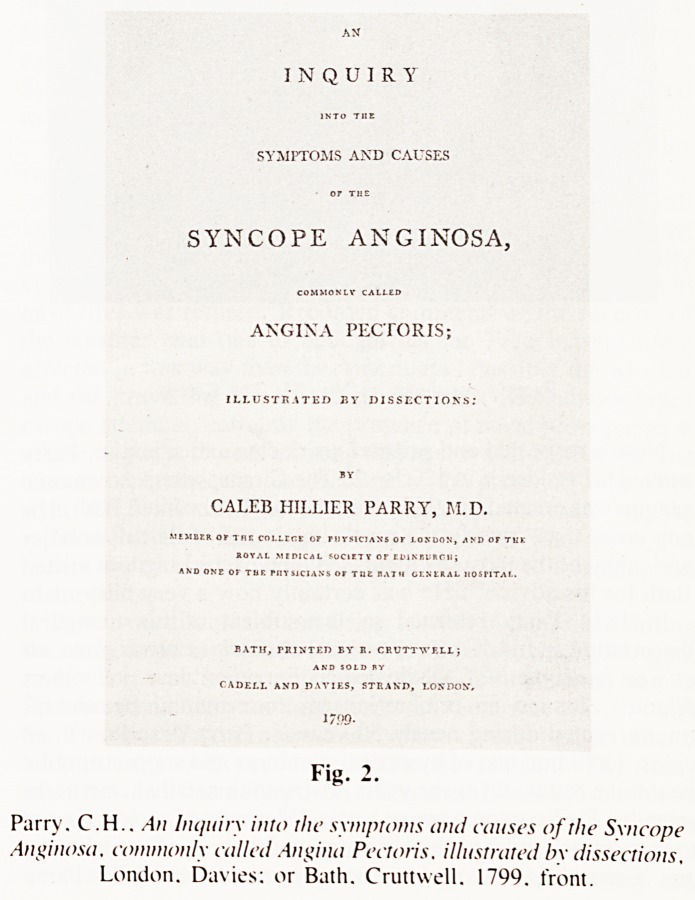


**Fig. 3. f3:**
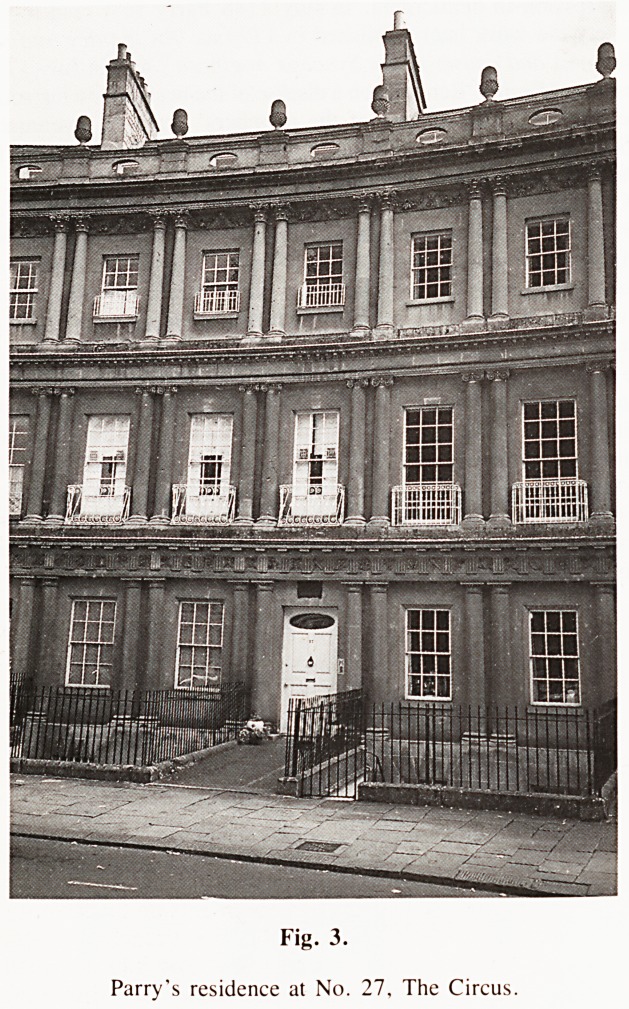


**Figure 4 f4:**